# Research on a Low-Cost, Low-Waste Method for Surface Flatness Improvement of WAAM Components Based on Tungsten Inert Gas Arc Remelting (TIGAR)

**DOI:** 10.3390/ma18010127

**Published:** 2024-12-31

**Authors:** Bo Zhao, Yuanlin Liu, Qingyuan Kang, Junjie Zhao, Guangyu Ma, Jie Wang

**Affiliations:** 1 School of Material Science and Engineering, Shandong Jianzhu University, Jinan 250101, China; 2023105125@stu.sdjzu.edu.cn (Y.L.); 202210103005@stu.sdjzu.edu.cn (Q.K.); zhaojj23@sdjzu.edu.cn (J.Z.); 2023105108@stu.sdjzu.edu.cn (G.M.); 202210103011@stu.sdjzu.edu.cn (J.W.); 2 Research Institute of Materials Reliability for Advanced Equipments, Shandong Jianzhu University, Jinan 250101, China

**Keywords:** tungsten inert gas arc remelting, wire-arc additive manufacturing, surface flatness improvement, robustness and adaptability, quantified surface roughness, tungsten inert gas arc welding (TIG)

## Abstract

Wire-arc additive manufacturing (WAAM) has fully empowered the design and manufacturing of metals with its unparalleled efficiency and flexibility. However, the process has relatively poor shape control capabilities, often requiring machining post-processing. This study explores a tungsten inert gas arc remelting (TIGAR) process to improve the surface flatness of WAAM components at a low cost and significantly reduce machining waste (up to 76%), which is crucial for the sustainable development of the process. The extent of surface improvement under different remelting currents was investigated. A detailed discussion was held on the mechanism by which the remelting arc, along with its molten pool, improves surface flatness. The robustness and adaptability of the process in a rough production environment were examined. And the impact of the process on the microstructure and hardness of the additive part’s surface was examined. Through systematic and quantitative analysis, it was found that within a certain range, the improvement effect on flatness increases with the increase in remelting current; when the remelting current reaches 80 A, it can significantly reduce the maximum height difference (65%) and the standard deviation of surface height (66%), and the remelting effect is uniform and reliable. TIGAR has a flattening effect on both the protrusions and depressions of the additive part’s surface. Proper overlap of remelted passes is crucial for the improvement of surface flatness. If the spacing of remelted passes is changed or the direction of remelting is adjusted, it is necessary to ensure the width of the molten pool to achieve sufficient overlap between adjacent remelted passes.

## 1. Introduction

Additive manufacturing is increasingly gaining traction within the manufacturing and engineering spheres [1]. This growing prominence can be attributed to its advantageous characteristics, notably its ability to facilitate unprecedented design freedom, enable on-demand production capabilities, and manufacture parts composed of various materials [2,3]. Among the myriad additive manufacturing techniques, wire-arc additive manufacturing (WAAM) employs a wire-feed system to precisely deliver metal wire at a predetermined rate, utilizing an electric arc as the heat source to melt the wire material [4]. This process facilitates the layer-by-layer deposition of molten metal onto a substrate, thereby incrementally building up the desired metallic component or structure [5]. This application of electric arc furnishes a synergistic combination of material efficiency and geometrical sophistication, markedly enhancing the capability to produce and repair large-scale metal parts within the current landscape of additive manufacturing research [6]. WAAM commonly utilizes robust and well-understood welding power sources such as tungsten inert gas arc welding (TIG), metal inert gas arc welding (MIG), cold metal transfer arc welding (CMT), etc., acclaimed for their unparalleled productivity and cost-effectiveness [7,8,9]. The capabilities of WAAM mentioned above pave the way for its extensive application potential.

However, WAAM often lags behind other additive manufacturing technologies (for example, selective laser melting) in shaping precision, which has been a limitation of its broader application [10]. “Shape control” and “property control” both pertain to the manufacturing quality of this process. When Scholars address the capability of WAAM to control the shape, they are actually discussing multifaceted issues encompassing (1) dimensional accuracy, referring to the deviation of part dimensions from the expected values caused by inaccuracies and instabilities in the actual deposited layer width and layer height, which may impact on the assembly and service performance [11]; (2) deformation caused by thermal and transformational stresses, which would degrade the service performance, fatigue life and even the structural integrity [12]; and (3) surface flatness and roughness of additively manufactured parts, which would affect the esthetics, fatigue life due to stress, friction and wear performance, and so on [13].

To resolve the first two challenges, namely achieving more precise dimensions of wire-arc additively manufactured parts and controlling the distortion arising during the deposition process, researchers implement a variety of strategies. These include the arc constriction method, the integration of weld seam tracking and closed-loop control systems, layer height compensation algorithms, optimization of processing parameters, the application of deposition boundary constraints, as well as many other similar methods used in welding [14]. Jia et al. [15] developed a novel compulsively constricted wire arc additive manufacturing method in which arc and droplets are ejected out of a narrow space to improve arc and droplet transfer behavior. This method can effectively control the geometry of each layer to obtain better performance and higher quality. Wang et al. [16] used a 3D scanner and cameras to achieve the detection of the width and reinforcement of the deposited layer during the fabrication of walled WAAM parts based on single-pass welding. They established an active disturbance rejection control (ADRC) strategy based on deep learning to adjust the welding current. Mu et al. [17] devised a compensation algorithm for forming accuracy, which compares the geometric data of each deposited layer with the CAD model. This algorithm optimizes and adjusts deposition parameters for the subsequent layer in real time, thereby correcting existing geometric errors, enhancing deposition precision, and addressing the deformation issues of the WAAM process. Diao [18] investigated the influence of process parameters on the weld bead morphology and droplet transition of CuCrZr alloy. Utilizing height data obtained from a distance sensor and applying compensation control algorithms, the dimensional stability of CuCrZr alloy WAAM components was enhanced. Yang et al. [19] proposed a “boundary constraint” method to address the issue of edge collapse of the additively manufactured thick-walled structure. Also, they employed a vertical cross-welding method, alternating between odd and even layers, to compensate for variations in layer height, which improved the forming accuracy and the overall deformation problem that accumulates during thick-walled structural manufacturing. Alhakeem et al. [20] developed an algorithm based on temperature variations and heat distribution, which generates the optimal deposition path, resulting in a 37% reduction in part deformation.

However, the issue of surface flatness or roughness in additive manufactured components arises from the inherent characteristics of the deposition process, which involves wire melting, metal (droplet) transfer, fluid flow within the molten pool, and solidification. The cross-sectional profile of each deposited layer is arc-shaped rather than square like a brick. Consequently, the layer-by-layer deposition beads naturally form a rough, undulating profile with alternating ridges and valleys on the surface of the WAAM parts. Many scholars in the research community today are particularly concerned with the microstructure and properties, relegating the surface flatness issue to post-processing machining and traditional deformation-control theories in the field of welding. In fact, traditional welding deformation control theories do not work well for this issue, and mechanical processing also faces the cost and environmental issues of cutting waste, as discussed in the next paragraph.

Spencer et al. [21] demonstrated that surface finish (similar to roughness or flatness) of additive manufactured parts can be enhanced using temperature control techniques. Xiong et al. [22] obtained a beautiful surface finish of the gas metal arc welding-based WAAM thin-walled structures with a combination of lower current (lower wire feed speed) and lower deposition rate. Nonetheless, the rough and wavy surface with alternating ridges and valleys is an intrinsic characteristic of the WAAM process, and can hardly be eliminated completely through parameter adjustments or process controlling. Consequently, post-processing remains necessary for additive manufacturing parts. Subtractive manufacturing is the most common post-processing technique, involving milling, grinding, and polishing to improve the surface flatness of WAAM components. Lopes et al. [23] employed milling to improve the surface flatness of HSLA (High-Strength Low-Alloy) thin-walled structures produced by WAAM. Researchers [24,25] also have developed hybrid manufacturing platforms that integrate additive and subtractive (milling) processes, capable of reducing surface roughness to 2 μm. However, the milling tools require a highly rigid holder or robotic arm, which is extremely costly, and it is challenging to process large-scale additive components, such as the runners of large hydro turbines [26] and architectural structures. Additionally, integrating additive and subtractive manufacturing processes can lead to material wastage during machining and incur additional processing time and costs. Although machining debris can be recycled, it is accompanied by extra processing costs and environmental concerns.

Certain researchers foresee employing “equivalent material” processing techniques, such as rolling and hammering, to induce surface plastic deformation. This approach aims both to improve the microstructure and to substantially reduce the surface roughness and stress of WAAM components. Fu et al. [27] integrated deposition and rolling processes in WAAM, employing a flat-rolling rig to compress the weld bead surface immediately after the deposition of each layer. Experimental results demonstrate that this approach achieves a relatively smooth surface while maintaining the deposition height of each layer. Liang et al. [28] employed ultrasonic impact on the freshly deposited bead positioned 40 mm behind the arc, which effectively reduced the internal stress within the aluminum alloy WAAM components. This approach also helps mitigate the accumulation error in multi-layer forming.

While utilizing rolling and ultrasonic impact on the freshly deposited bead are crucial for controlling the formation of the top surface of the component during the additive manufacturing, i.e., the fresh and relatively hot surfaces not very long after the arc burning, they do not address the flatness of the sides of the additive component, which typically necessitates additional machining, leading to material waste and increased costs. Honnige et al. [29] employed vertical and lateral rolling techniques in the fabrication of 2319 aluminum alloy using wire arc additive manufacturing and investigated their effects on the residual stress, deformation, and hardness of the parts. The results indicated a significant reduction in residual stress and deformation, accompanied by an increase in hardness. However, because the sides had solidified and cooled, resulting in significant resistance to plastic deformation, the effectiveness of side rolling is limited. The rolling process only slightly smooths the surface, and the rolled surface still exhibits noticeable pits. For steel additive components with higher strength, it is even more challenging to improve surface flatness through side rolling. Xie et al. [30] reduced the surface flatness of the side of a single-layer, multi-pass, thin-walled additive manufacturing part through a hot rolling process. With the addition of a horizontal rolling mechanism, the maximum width absolute error on the side was reduced from 0.45 mm to 0.12 mm. But the hot rolling equipment increases complexity and cost, as well as the energy consumption required for heating and applying force.

Chen et al. [4] employed laser surface remelting on wire-arc additive manufacturing components, combining the high deposition rate of WAAM with the energy-controllable advantages of laser heating. Xue et al. [31] established a simulation model for heat transfer and melt pool flow during the laser remelting process, and experimentally verified the effectiveness of the remelting process: when the laser power is at 900 W, the surface roughness is relatively low, reducing by 60.2% compared to the unrefined parts. Ko and colleagues [32], based on the principles of the laser remelting process, adjusted parameters such as feed rate and beam power, and were able to reduce surface roughness significantly. Despite the advantage of laser remelting in enhancing the surface morphology, its significantly higher costs and increased equipment complexity compared to arc processes may limit its widespread application. Comparable to the laser remelting process, the tungsten inert gas arc, activated by a TIG welding power source and shielded in purified argon or helium gas, is capable of remelting parts well beyond the operational range of a machine tool while avoiding debris generation. The tungsten inert gas arc offers several advantages, including low heat input (which minimizes the heat-affected zone), effective protection of the molten pool through inert gas, and reduced costs. The equipment required for this process, namely the TIG welding power source priced below $400, is widely available and commonly used. Factories with WAAM production lines may already be equipped with TIG welding powers. However, researchers often overlook the exceptional capabilities of the simple arc process.

This paper employed the tungsten inert gas arc remelting (TIGAR) process as a highly cost-effective method for enhancing the surface flatness of WAAM parts, aiming to significantly reduce or even eliminate the need for machining. Encouraging results were obtained. In this study, a chromatic confocal microscope (CCM) was used to reconstruct the digital three-dimensional surfaces of WAAM parts and scientifically evaluate the improvement in surface flatness after TIGAR with varying welding currents. Additionally, experiments were conducted under various remelting spacings and directions, thereby investigating the robustness of this process and validating its adaptability under practical, less controlled production conditions. The effectiveness of the process in reducing the amount of subsequent machining (if necessary) was further evaluated. At last, the hardness and grain size of the remelted areas were measured, and a preliminary assessment was conducted on how the additional thermal cycles introduced during remelting affect the difficulty of subsequent machining and the service performance of the WAAM components.

## 2. Materials and Methods

**Figure 1:**
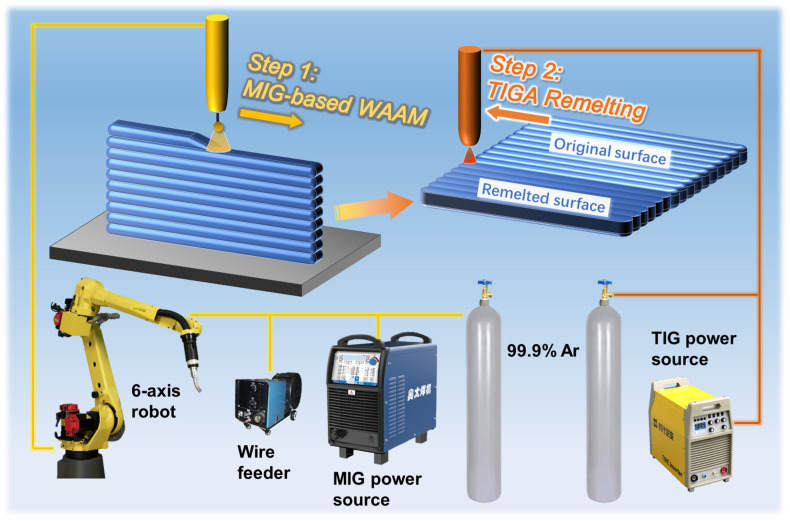
Schematic of the WAAM process for thin-walled parts and the TIGAR process.

A robotic MIG-based wire-arc additive manufacturing system, comprising a Pulse MIG-350RP welder (AOTAI, Jinan, China), an ESS-500 wire feeder (AOTAI, Jinan, China), and a M-10iD/12 robot with a R-30iB Mate Plus controller (FANUC, Yamanashi, Japan), was employed to fabricate thin-walled metallic structures using a multi-layer, single-pass deposition program. Layer-by-layer deposition was conducted on a Q235 mild steel (according to GB/T 700–2006 [33]) substrate utilizing a 1.2 mm diameter 316 L stainless steel (according to GB/T 29713–2013 [34]) filler wire, as illustrated on the left side of Figure 1 (Step 1). The chemical elemental composition of the filler wire is detailed in Table 1. Argon with 99.9% purity (at a shield gas flow rate of 15 L/min) was released from the shielding gas nozzle encircling the wire to safeguard the droplets and the melt pool from oxidation. The principal parameters of the additive manufacturing process encompass welding current, arc voltage, travel speed, and interlayer cooling time. Variations in these parameters influence the morphology and mechanical properties of the deposited parts. Drawing upon the relevant literature [35,36] and a substantial amount of experimental data, a set of well-formed WAAM process parameters had been selected, as detailed in Table 2.

After the additive manufacturing was completed, the additive part was cut off from the substrate, and the TIGAR process was applied to the undulating surface of the overlapping deposited layers on the side of the additive part, as shown on the right side of Figure 1 (Step 3). To facilitate the control of the remelting arc’s position, a welding robot was utilized to manipulate the welding torch. Considering the following points, we first investigated the effect of remelting currents on the improvement of surface flatness; for TIG arcs, current is the most critical parameter, with other parameters needing to match the current and having a limited range of adjustability. The arc voltage is related to the arc length, which is subject to many restrictions in the actual usage environment and is also disturbed by the uneven surface. For a low-cost, energy-efficient surface flatness improvement technology, the remelting current is generally low. So, to ensure qualified weld formation, there is an upper limit to the remelting speed, which cannot be increased without restriction. Therefore, we selected a remelting speed of 1 mm/s, which could form the most stable molten pool in our preliminary experiments. Other parameters, such as the shielding gas flow rate (argon with a purity of 99.9%, 12 L/min), are the common process parameters.

Different remelting currents (Test A1—A4: 40 A, 60 A, 80 A, and 100 A) were used, and the arc traveled along the direction of the as-deposited additive weld beads. The arc voltage for remelting and the TIG-welding-torch travel speed were set as described in Table 3.

Based on a conceptual understanding of the effects and mechanisms of arc remelting, it can be inferred that the remelting arc can flatten the “ridges” on the undulating side surface of the overlapping deposited layers of additive-manufactured components. Therefore, positioning the centerline of the torch directly through these ridges may achieve optimal results. However, in practical manufacturing environments, the absence of robots, highly skilled workers, precise and convenient jig and fixture systems, and effective quality management systems, as well as the over-emphasis on production efficiency, often means that it is challenging to ensure that the arc is perfectly aligned with the ridges throughout the remelting process time. Therefore, this paper further investigated the effects of different remelting spacings (distance of the adjacent remelting pass centers) and remelting directions (the remelting direction is at a certain angle relative to the additive manufacturing deposition direction) by deliberately misaligning the remelting arc with the ridge. It simulated rough production conditions without quality management and verified the robustness of the TIGAR process and its adaptability to the practical factory production environment.

The optimal remelting current 80 A was selected based on the results of Test A1–A4. This will be detailed in Section 3.1, in which you will find that as the remelting current increases, the flatness gradually improves, with the improvement at 80 A being extremely significant, while further increasing the remelting current (to 100 A) results in no significant change in flatness. Clearly, under the conditions of this study, 80 A is more energy-efficient than 100 A and is more suitable as the remelting current. Thus, Tests B1 and B2 were conducted and compared with the results of Test A3 to explore the impact of remelting spacing (3 mm, 4 mm, and 5 mm, respectively) on the improvement of surface flatness. Additionally, Tests C1 and C2 were also performed and compared with the results of Test A3 to investigate the effect of remelting direction (the remelting direction is at 0°, 45°, and 90° relative to the additive manufacturing deposition direction, respectively) on the improvement of surface flatness. The TIGAR process parameters for the aforementioned experiments are shown in Table 3.

A KC-X1000 CCM (KathMatic, Nanjing, China) was employed to precisely reconstruct and analyze the 3-D profiles of the specimen surfaces. Utilizing the 3-D reconstruction data, the mean deviation and standard deviation of the measured areas’ contours (height or the z-axis coordinates) were calculated. The surface flatness before and after remelting was subsequently evaluated. The consistency of the remelting process in enhancing the surface flatness of WAAM parts was evaluated in conjunction with the flatness data of other measured areas under identical parameters.

Although the TIGAR process improved the surface morphology, subsequent machining may still be required for applications with high surface precision and roughness demands. In consideration of the aforementioned application scenarios, the Vickers hardness and microstructure of additively manufactured parts’ surfaces after TIGAR treatment were tested to preliminarily assess the difficulty of subsequent machining and service performance. A fully automatic microhardness tester was used to measure the hardness of the remelted and non-remelted areas, with a working load of 50 N and a loading time of 10 s. Ten random test points were selected in each tested area. The cross-section of the samples was prepared using wire electrical discharge machining (WEDM), and these cross-sections were ground and polished. The surfaces were etched with aqua regia solution for 55 s, and the grain sizes in the remelted and non-remelted zones were observed using a MERLIN Compact (Zeiss, Oberkochen, Germany) scanning electron microscope (SEM). Additionally, an Oxford X-Max (Oxford Instruments, Oxford, UK) energy dispersive spectrometer (EDS) was employed to determine the distribution and content of elements.

## 3. Results and Discussion

### 3.1. Surface Flatness Improvement Under Different TIGAR Currents

#### 3.1.1. Surface Morphology Before and After Remelting

**Figure 2:**
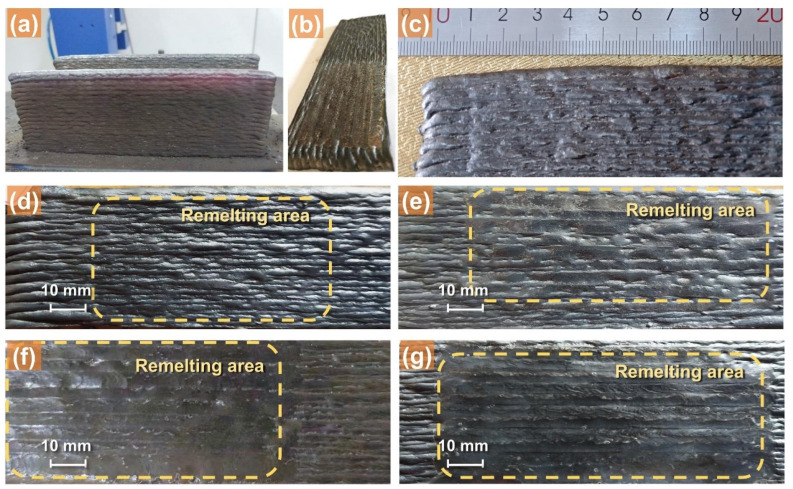
The WAAM thin-walled structures before (**a**) and after (**b**) the TIGAR process; the surface morphology of WAAM; (**c**) remelted zones under remelting current 40 A (**d**), 60 A (**e**), 80 A (**f**), and 100 A (**g**).

Figure 2a illustrates the wire-arc additively manufactured wall-shaped structures, while Figure 2b depicts an additive structure after the TIGAR process. Figure 2c–g show the surface morphology of the as-deposited additive parts and their remelted zones under different TIGAR currents. The TIGAR process parameters are as mentioned in Section 2, the paragraph followed by Table 2. The remelting direction is consistent with the MIG-torch’s travel direction during additive manufacturing. The spacing between centers of adjacent remelting passes is set to 4 mm to match the layer height of the WAAM process, ensuring that the tungsten electrode aligns with the ridges of the undulating surfaces of additively manufactured structures.

The effect of the remelting process on the surface morphology can be easily identified by visual inspection. It can be observed in Figure 2c that the non-remelting surface of the WAAM structure exhibits significant undulations, with distinct “ridges” formed by each deposition layer and noticeable “valleys” between adjacent layers, clearly defining the boundaries of each layer. When the remelting current is 40 A, the remelting passe is not obvious, and adjacent remelting passes do not overlap, as shown in Figure 2d. When the remelting current increases from 40 A to 60 A, some remelted welds can be clearly distinguished, and their surfaces become smoother, as shown in Figure 2e. Although the adjacent remelted welds do not fully overlap, the gaps between them are reduce. The surface flatness seems to change slightly, and some “ridges” are eliminated. When the remelting current increases to 80 A, the remelted welds completely overlap, and the apparent ridges and valleys nearly disappear, resulting in a visibly smoother and significantly flattened surface, as shown in Figure 2f. Furthermore, no visible defects such as pores and cracks are found in the entire remelted surface. When the remelting current is further increased to 100 A, the surface remains very flat, as shown in Figure 2g. But compared to 80 A, there are no visibly noticeable changes in surface morphology improvement.

Based on the visual observations with the naked eye, above, it can be preliminarily concluded that TIGAR can smooth the surface of WAAM components at specific currents. To assess more precisely the remelting effect, the authors quantified the surface flatness under the aforementioned conditions.

**Figure 3:**
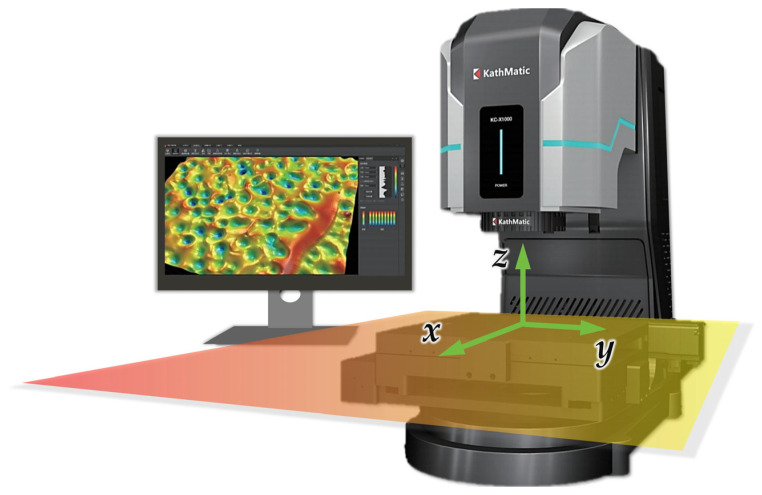
Schematic of the laser confocal microscopy and measurement coordinate system.

Traditionally, a probe profilometer is commonly employed to measure the surface “roughness” of a workpiece. However, the height measurement range of a probe profilometer is typically small, whereas the surface undulations of the WAAM components in this experiment usually reach several millimeters, exceeding the scope of what is generally considered surface roughness (this is also the reason why the authors use the word “flatness” instead of “roughness” in this study). Furthermore, due to probe tip wear or a specific taper, the probe tip may not reach the bottom of relatively small pits, leading to a decreased variation in the measured fluctuation height. Even with the use of the most advanced probe profilometer, it remains challenging to evaluate the flatness of an entire surface as it is more suitable for measuring line roughness. Hence, the chromatic confocal microscope introduced in Section 2 was employed to perform a three-dimensional reconstruction of the remelted and non-remelted surfaces, as illustrated in Figure 3, for a more comprehensive analysis of the surface morphology. The technique offers enhanced precision in detecting complex and steep topographies. The operating platform of the microscope is situated in the *x*–*y* plane, with the normal direction aligned along the *z*-axis, as shown in Figure 3. Each measured area has a geometry range of 10,000 μm × 10,000 μm in the *x*–*y* plane, and progressive scanning is conducted within these areas. Each scanning line is oriented along the *y* direction in the figure, with a line spacing of 20 μm, and test points are sampled every 20 μm along every scanning line (along the *x* direction). Each measured area contains 500 × 500 = 250,000 test points, and every point’s height (*z*-axis coordinate) was detected via the principle of chromatic aberration confocal microscopy [37]. Based on all the points’ heights along with their coordinates within the *x*–*y* plane, a 3-D reconstructed contours of the measured areas can be established.

**Figure 4:**
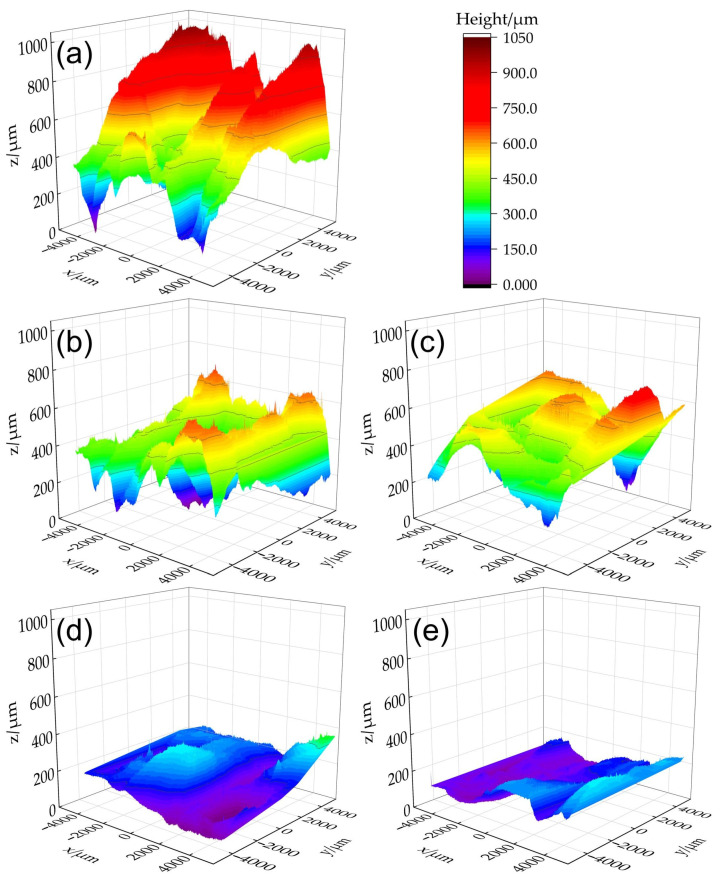
Three-dimensional reconstructed contours of the typically measured areas on the surface of (**a**) the WAAM component and within the remelting areas with remelting currents of (**b**) 40 A, (**c**) 60 A, (**d**) 80 A, and (**e**) 100 A.

Figure 4 presents the three-dimensional reconstructed contours of the typically measured areas on the surfaces of the as-deposited WAAM component and within the remelted zones. For ease of observation and comparison, the height of the lowest point on each measured area is standardized to *z* = 0. It is clearly shown in Figure 4a that there are ridges and the valleys between them within the non-remelted measured area. When the remelting currents are 40 A (Figure 4b) and 60 A (Figure 4c), the maximum heights of the measured areas are 698.4 μm or 762.34 μm, respectively, which are 33% and 26% lower than the maximum height of 1024.8 μm observed in the non-remelted area (Figure 4a). Most sub-areas exhibit heights between 0 and 400 μm, with only a small portion of sub-areas exceeding 600 μm. Although the height of the ridges has been significantly reduced, the ridges and the valleys are still relatively pronounced. With further increases in current to 80 A (Figure 4d) and 100 A (Figure 4e), the maximum heights are reduced to 362.82 μm and 301.43 μm, respectively, representing decreases of 65% and 71% compared to the non-remelted area. Most sub-areas are located in the deep blue and purple regions ranging from 0 to 200 μm, and the maximum height is roughly halved even compared to the 40 A and 60 A results, indicating a significantly flattened surface. The measuring data confirm the visual observations in Figure 2.

#### 3.1.2. Mechanism for Flatness Improvement

Remelting causes remarkable changes in the surface flatness of additive manufactured structures, as mentioned above. Obviously, this phenomenon can be attributed to the formation of a molten pool during the remelting process and the metal fluid dynamics within this pool. As the remelting current increases, with the travel speed and arc voltage remaining constant, there is a corresponding increase in heat input. This results in an expansion of the molten pool’s volume, which is generated by the remelting arc, as well as an extension of the area over which the fluid metal can flow. Concurrently, the metal within the molten pool remains above the liquidus temperature for a longer period, enabling it to flow and spread more adequately under the influence of various forces. This provides more time for the surface undulations to be flattened. Consequently, when employing higher remelting currents, such as 80 A and 100 A, the enhancement in surface flatness becomes more pronounced.

**Figure 5:**
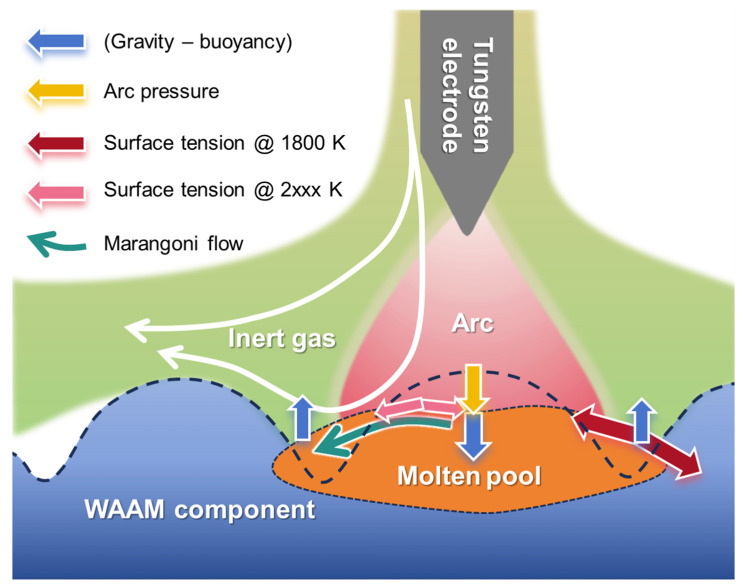
Schematic of the forces and fluid flow pattern in the molten pool during remelting.

During an adequate flow time, the stress conditions within the molten pool determine whether its flow will cause ridges to subside and valleys to be filled. Under the influence of a tungsten inert gas arc, the forces acting on and the fluid flow phenomena within the molten pool are depicted in Figure 5. Given that the remelting current is relatively low (*I* ≤ 100 A) and the molten pool will only have a slightly deformed surface, the electromagnetic force and arc shear stress viscous, a viscous drag force induced from tangential velocity difference between plasma and molten pool are minimal [38,39]. The shape of the molten pool’s upper surface is primarily influenced by the arc pressure, gravity (and buoyancy), surface tension, and the Marangoni flow associated with surface tension gradients [40]. Ignoring the static pressure variation due to changes in arc diameter when the arc length is short (a TIG arc with current *I* ≤ 100 A; arc voltage *U* = 14 V), the typical equation for arc pressure *P*_A_ can be detailed as follows:

(1) $$P_{A}=\frac{\mu_{m}{Ι}^{2}}{8\pi^{2}\sigma_{A}^{2}}\exp(-\frac{r^{2}}{2\sigma_{A}^{2}})$$

where $\mu_{m}$ represents the permeability, *I* is the current of remelting arc, r signifies distance from a given point to the arc center, and $\sigma_{A}$ indicates the current distribution parameter.

According to Equation (1), *P*_A_ is proportional to the square of *I*, and as σ_A_ increases (σ_A_ > 0), *P*_A_ decreases. Under constant environmental pressure and arc composition, σ_A_ increases with *I*, but obviously the rate of increase in σ_A_ is much less than that in *I* [41]. Consequently, *P*_A_ generally increases with *I*. When the surface of the molten pool rises, such as at the ridge that has just been melted by the remelting arc, the arc pressure is directed inward, flattening the surface of the pool.

Usually, the gravitational force, *Gb*, on the surface of the molten pool is expressed as a function of the surface morphology of the molten pool, that is,

(2) Gb=−ρgϑ

where ρ represents the density of molten metal, g indicates the gravitational acceleration, and ϑ is the surface morphology expressed as follows,

(3) ϑ=z(x,y)

Equation (2) expresses a trend where the gravitational force on areas above the baseline acts downward, while on areas below the baseline, the gravitational force acts upward. This expression of gravity actually encompasses the effect of buoyancy. When the surface of the molten pool bulges, such as at the ridge where the pool has just been heated and melted, gravity exerts an effect that promotes the central surface of the molten pool to flow downward and flatten.

For the entire molten pool, surface tension is a crucial force in maintaining the bulging liquid surface [42]. Numerous studies have indicated that the surface tension of low-sulfur stainless steel decreases as the temperature rises, particularly in the interval exceeding 1800 K [43,44,45,46]. Additionally, the liquidus temperature of 316 L stainless steel is above 1700 K [47], indicating that in the majority of the liquid temperature range and the majority of subareas of the molten pool surface, the surface tension temperature gradient is negative. As the average temperature rises with a higher remelting current, the average surface tension of the molten pool diminishes, and its obstructive influence on the molten pool’s spread may become less significant at high current levels.

Furthermore, under a negative surface tension temperature gradient, the surface tension in the high-temperature center of the molten pool will be lower than that in the areas at 1800 K temperature (usually located at the edges of the molten pool [48,49]), as shown in Figure 5. The negative surface tension temperature gradient drives Marangoni flow from the center to the periphery of the molten pool, facilitating the flattening of the convex surface of the molten pool. With increasing current, both the temperature gradient and the central temperature of the molten pool may rise, leading to an enhanced Marangoni effect. As analyzed above, gravity, arc pressure, and Marangoni flow facilitate the spreading of the molten pool along the ridge, while surface tension impedes this spread. As the current increases, arc pressure and the Marangoni effect are intensified, whereas the hindrance of surface tension becomes relatively limited. Gravity remains unaffected by the current and is solely dependent on the molten pool’s shape. Thus, higher current levels are more favorable for flattening the surface of the WAAM components, which elucidates the observations depicted in Figure 4.

#### 3.1.3. Quantified Surface Flatness

**Figure 6:**
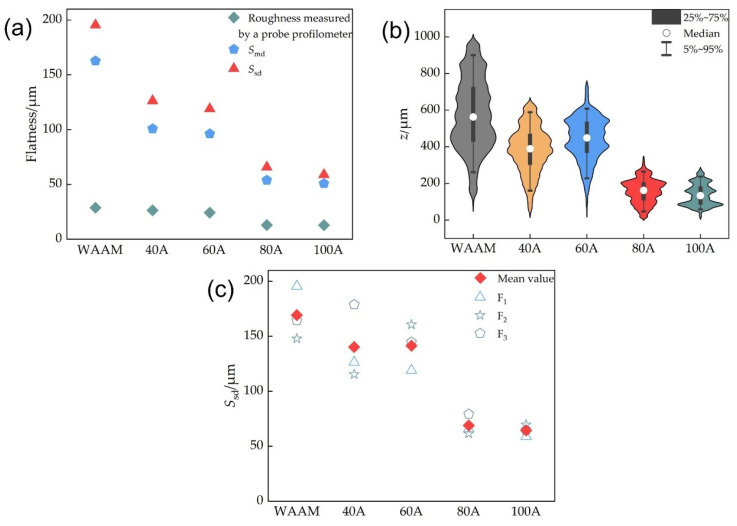
Characterization of surface morphology of measured areas under different remelting currents: (**a**) quantified flatness—*S*_sd_, *S*_md_, and the “roughness” measurements via a probe profilometer; (**b**) violin plots illustrating height distribution; (**c**) *S*_sd_ values on three randomly selected areas.

To further quantify the flatness of the remelted surface, the standard deviation (*S*_sd_) and arithmetic mean deviation (*S*_md_) of the heights of all test points on the measured areas in Figure 4 were calculated based on the confocal measurement results, as illustrated in Figure 6. The standard deviation *S*_sd_ is calculated as the square root of the average of the squared differences between each test point’s height and the mean height of all points of the area, which is determined by Equation (4). The arithmetic mean deviation *S*_md_ is computed as the average of the absolute differences between each test point’s height and the average height, as expressed in Equation (5).

(4) $$S_{sd}=\sqrt{\frac{1}{\left(M-1)(N-1\right)}\sum_{i=1}^{M}\sum_{j=1}^{N}(z_{ij}-\bar{z}{)}^{2}}$$

(5) $$S_{md}=\frac{1}{\left(M-1)(N-1\right)}\sum_{i=1}^{M}\sum_{j=1}^{N}\left|z_{ij}-\bar{z}\right|$$

Here, *i* and *j* represent the indices of the measured points, where *i* ranges from 1 to *M* and *j* ranges from 1 to *N*. *M* represents the number of points in each row when scanning with the confocal microscope, and *N* is the number of rows containing the points. In this experiment, both *M* and *N* are 500 as mentioned in Section 2. The divisors (*M −* 1) and (*N −* 1) are used because, relative to the entire surface, the measured area represents only a portion of the sample. Despite the sufficiently fine 20 μm point spacing, the height data collected is considered representative of the sample, hence (*M* − 1) and (*N* − 1) are utilized to derive more significant roughness metrics. *z_ij_* represents the height, or the *z*-coordinate of the No. *i* point in *j* row. And $\bar{z}$ corresponds to the average heigh, which is defined as follows,

(6) $$\bar{z}=\frac{1}{MN}\iint_{\Omega }z\left(x,y\right)dxdy$$

where *Ω* represents the measured area. However, it is not possible to describe the shape of the measured area *Ω* with an integrable elementary function. With a sufficiently dense sampling of points, this research used the average height of the aforementioned 250,000 measured points as an effective approximation of the average height for the entire measured area. Therefore, $\bar{z}$ can be determined using Equation (7),

(7) $$\bar{z}=\frac{1}{MN}\sum_{i=1}^{M}\sum_{j=1}^{N}z_{ij}$$

Figure 6a illustrates the variations in standard deviation *S*_sd_ and arithmetic mean deviation *S*_md_ values with different remelting currents. Both *S*_sd_ and S_md_ assess the surface flatness and the extent of deviation from the average height. Both parameters exhibit a consistent trend, decreasing with increasing remelting currents, thereby indicating an enhancement in surface topography. However, the “roughness” values measured by a conventional probe profilometer are notably lower compared to the maximum height differences shown in Figure 4. As discussed in Section 3.1, the roughness values are underestimated.

At each current setting, the *S*_sd_ values are marginally higher than *S*_md_, which is potentially influenced by a few outlier points distant from the average height. The *S*_sd_ metric accentuates the impact of localized uneven areas on the flatness—even minor surface irregularities can markedly amplify *S*_sd_. In practical applications, the flatness of a certain specific location can influence the part’s qualification. Compared to *S*_md_, the *S*_sd_ value holds a greater significance in evaluating the serviceability of parts and in determining the necessary depth of milling required for subsequent post-processing. Therefore, the authors believe that adopting the standard deviation of the height values of the measured areas as a quantification standard for surface flatness is a more responsible approach. Thus, subsequent analyses will focus on the standard deviation *S*_sd_. In Figure 6a, the improvements in *S*_sd_ for 40 A and 60 A are comparable, with reductions of 35% and 39%, respectively, relative to the as-deposited surface without remelting, indicating a notable enhancement. As the remelting current increases to 80 A and 100 A, *S*_sd_ values decrease by 66% and 70%, respectively, with similar improvements in surface topography. Moreover, from 40 A to 100 A of current, the reduction in standard deviation is greater than that of the arithmetic mean deviation. Based on the previous discussion, it may be concluded that the increase in remelting current is more effective in eliminating local sharp irregularities. Future work may include more specific studies on a broader range of surface topographies to substantiate this hypothesis.

The violin plot illustrates the probability density distribution of surface heights for each parameter. As depicted in Figure 6b, both 80 A and 100 A of remelting currents significantly diminish the disparity in maximum undulation height, lower the median height (relative to the lowest point), and result in a narrower height distribution; hence, both *S*_sd_ and *S*_md_ are markedly reduced. For the measured areas subjected to remelting currents of 80 A and 100 A, the height distribution ranges of points with larger deviations from the median height value (points with heights below the 5th percentile or above the 95th percentile) are similar, and significantly reduced compared to those of the areas remelted with currents of 40 A and 60 A. This observation lends some credence to the previous hypothesis.

Given the diversity of the WAAM surface morphology, the authors wonder whether the effect of TIGAR may vary across different areas. So, three measured areas were randomly selected in the remelted zone with each remelting current, and their *S*_sd_ values were measured and calculated to determine whether the improvement effect of the remelting process on the surface morphology is uniform. Figure 6c demonstrates that the maximum discrepancy among *S*_sd_ values on the three measured areas named F_1_, F_2_, and F_3_ for 80 A remelting current is only 17.8 μm, and the variation for 100 A is even less (10.8 μm). This indicates that the improvement effect of the TIGAR method on the surface flatness of the additive manufactured parts under a current of 80 A or 100 A is relatively stable, and the improvement capability is quite reliable. Subsequently, any one of the randomly chosen areas could be selected for further study. However, as the remelting current decreases, the range of *S*_sd_ values gradually expands. The *S*_sd_ values of the three measured areas in the remelted zone with the current of 40 A show relatively larger variations of (up to 63.4 μm), indicating that low-current remelting not only has poor improvement effects on the surface flatness of the WAAM components but also lacks stability.

#### 3.1.4. Quantity of Cutting Waste After Remelting

As previously discussed, the arc remelting method can substantially reduce the surface flatness of arc WAAM components. While this process is suitable for applications where surface flatness requirements are not stringent, many other applications still necessitate mechanical post-processing.

The following discussion addresses the reduction in cutting waste and cutting time achievable after the TIGAR process. To simplify the analysis, it is assumed that the additive manufacturing parameters and the entire additive-subtractive manufacturing process specifications have been optimally designed based on the aforementioned surface topography and flatness data. Consequently, by merely removing the material on the additive part’s surface that is above the lowest point, the design dimensions of the part can be precisely achieved. Under this assumption, the plane where the lowest point of the part’s surface resides becomes the new surface after milling, which corresponds to the minimum milling depth required.

The volume between the as-deposited surface of the additive part and the new surface after milling constitutes the minimum quantity of the metal to be machined (milled), which can be calculated using the double integral provided in Equation (8).

(8) $$V=\iint_{\Omega }(z\left(x,y\right)-z_{min})dxdy$$

where *z*_min_ indicates the minimum value of the surface profile. Again, the difficulties encountered in solving Equation (6) reemerge. To overcome this, the authors employ the following formula to avoid the necessity of describing the function of the surface shape.

(9) $$V=\frac{100}{250000}\sum_{i=1}^{M}\sum_{j=1}^{N}{(z}_{ij}-z_{min})$$

With a sufficiently dense sampling of points (250,000 points in a 10 mm × 10 mm measured area), the double integral is converted into the sum of the products of the heights of all test points and their respective control areas based on the principles of calculus.

**Figure 7:**
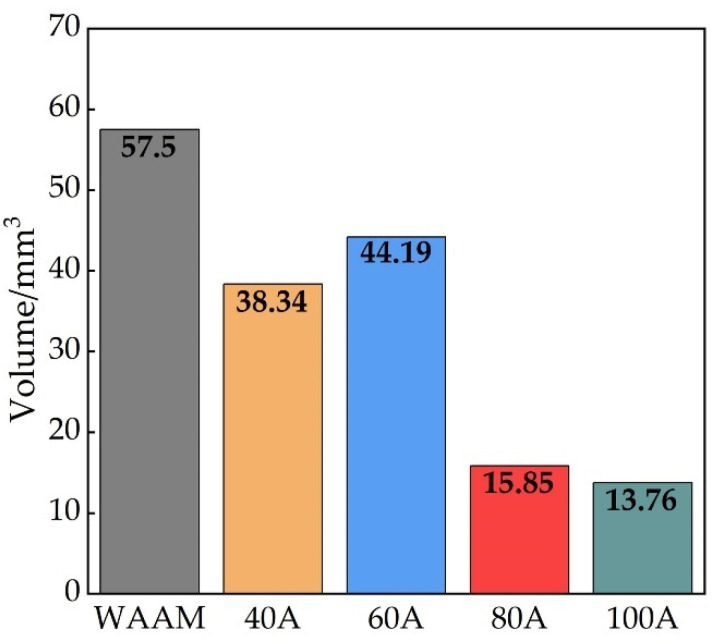
Minimal quantities of milling for the measured areas under varying remelting currents.

The minimal quantities of cutting waste calculated utilizing Equation (9) are as depicted in Figure 7. The cutting waste for the measured area of the as-deposited surface fabricated by the WAAM process is 57.50 mm^3^. In contrast, the cutting waste for measured areas of remelted zones at TIGAR currents of 40 A, 60 A, 80 A, and 100 A are 38.34 mm^3^, 44.19 mm^3^, 15.85 mm^3^, and 13.76 mm^3^, respectively. It is worth emphasizing that these are data measured only within a local area of 10 mm × 10 mm. Notably, at currents of 80 A and 100 A, the cutting waste can be reduced by up to 72% and 76%, respectively. This reduction significantly contributes to cost savings and enhances environmental sustainability.

The machining time required for milling is influenced by a combination of factors, including cutting depth, milling cutter diameter, cutting speed (or spindle rotation speed), and feed rate. These process parameters are, in turn, affected by various factors such as surface morphology, material strength, tool form, and tool material, making the calculation of specific machining times quite complex. In this study, the typical cutting depth (also called “axis feed” in some papers) *a*_p_ for post-processing of stainless steel additive parts (within the range of 0.3 mm–0.75 mm), as reported in the literature [50,51,52,53], were selected to simplify the analysis. Based on the height data in Figure 6b, it can be seen that after TIGAR treatment, the number of milling layers is reduced by at least one. Especially under the remelting currents of 80 A and 100 A, the number of cutting layers could potentially be halved, suggesting a significant reduction in cutting time. Additionally, for 3D-printed architectural load-bearing structures, urban art installations, and the like, it is even possible to eliminate the need for machining processes. Therefore, this approach reduces costs and offers economic benefits.

### 3.2. Changes in Surface Characteristics with Various Remelting Spacing

**Figure 8:**
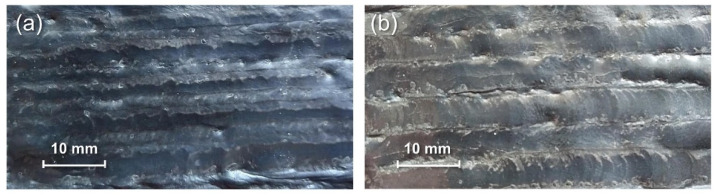
Surface morphology of remelted zones with remelting spacings of (**a**) 3 mm and (**b**) 5 mm.

**Figure 9:**
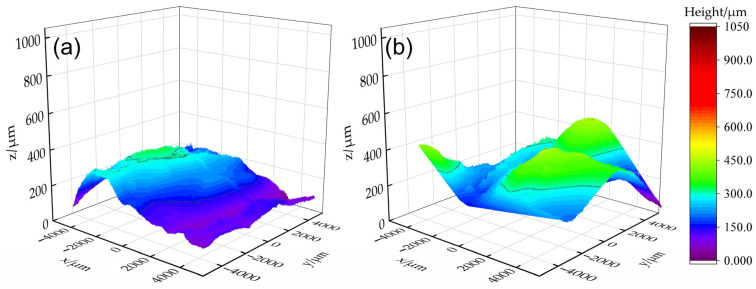
Three-dimensional reconstructed contours of the typically measured areas with remelting spacings of (**a**) 3 mm and (**b**) 5 mm.

In Section 3.1, the remelting spacing (distance of the adjacent remelting pass centers) of 4 mm is close to the layer height of the original additive part, allowing the axis of the tungsten electrode to be aligned with the “ridge” for remelting. As described in Section 2, to investigate the adaptability and robustness of the TIGAR process, the authors simulated the situation where the remelting arc is not aligned with the “ridge” in a rough production environment, thus conducted TIGAR experiments at different remelting spacings. When the remelting spacings were 3 mm and 5 mm, respectively (with a remelting current of 80 A and other process parameters the same as in previous experiments, i.e., arc voltage of 14 V and remelting speed of 1 mm/s), the surface morphology is shown in Figure 8a,b. It can be observed that when the remelting spacing is 3 mm, the adjacent remelted passes overlap sufficiently, with no cracks or other defects detected. And the surface morphology is close to that of the remelting zone with a 4 mm remelting spacing (Figure 2f). At a remelting spacing of 5 mm, a small portion of the adjacent remelted passes do not fully overlap, and there are pits and “rough” areas between the remelted passes, resulting in a poorer surface morphology compared to the 3 mm and 4 mm specimens. But it still shows a significant improvement compared to the as-deposited surface of the additive parts. The corresponding three-dimensional reconstructions of the typically measured areas reveal that the surface height distribution for the 3 mm (Figure 9a) and 4 mm (Figure 4d) remelting spacings are quite similar, with most heights in the blue and purple (<200 μm) regions, and only a few locations in the light green (300–350 μm) region. In contrast, the surface of the 5 mm (Figure 9b) specimen has a considerable portion in the light blue and green regions (300–450 μm), indicating a slight increase in surface undulation compared to the former two. However, compared to the as-deposited surface without remelting, the maximum height is significantly reduced.

**Figure 10:**
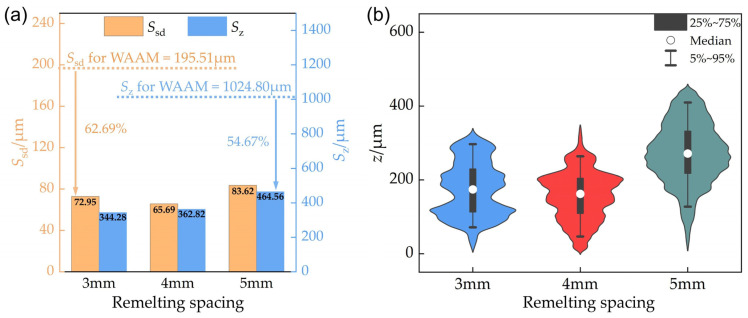
Characterization of surface morphology of measured areas with different remelting spacing: (**a**) standard deviation of height (*S*_sd_) and maximum height difference (*S*_z_); (**b**) violin plots illustrating height distribution.

The standard deviation of height *S*_sd_ and the maximum height difference *S*_z_ of the measured areas in the figure above are shown in Figure 10a. When the remelting spacing is 3 mm, 4 mm, and 5 mm, the standard deviations *S*_sd_ are 72.95 μm, 65.69 μm, and 83.62 μm, respectively, which are reduced by 62.69%, 66.40%, and 57.23% compared to the as-deposited area without remelting. The maximum height differences *S*_z_ for the areas with remelting spacings of 3 mm, 4 mm, and 5 mm are 344.28 μm, 362.82 μm, and 464.56 μm, respectively, which are reduced by 66.41%, 64.60%, and 54.67% compared to the non-remelted area, showing a significant reduction. From Figure 10b, it can be seen that the height distribution of the three measured areas all exhibit a violin shape with a wide middle and narrow ends, with the medians close to the center of the “violin”. The height difference in the 25–75% percentiles is a critical measure in statistics, commonly referred to as the interquartile range (IQR) in height. The IQRs in height are 116.64 μm (3 mm), 95.66 μm (4 mm), and 114.53 μm (5 mm), respectively, showing no significant difference. The above results indicate that within a certain range of remelting spacing, the TIGAR process can significantly improve the surface flatness of additive parts, demonstrating the robustness of this process.

However, it is worth noting that the median height value of the measured area with remelting spacing of 5 mm is higher, and the length of the 5–95% percentile interval is 281.69 μm, which is 64.17 μm higher than the 4 mm result. This is an increase of about 22.8%. Moreover, the distance between the maximum and minimum values is quite far—the maximum height difference *S*_z_ of the 5 mm specimen is increased by 120.28 μm and 101.74 μm compared to the measured areas with remelting spacings of 3 mm and 4 mm, respectively. This indicates that the 5 mm specimen has more pronounced peaks and valleys that are a bit far from the median. Observing Figure 8b, it can be speculated that these locations with poor flatness may be in the areas between the remelted passes that are not well overlapped. When the distance between the centers of adjacent remelted passes is too far, there is no good overlap between the remelted passes. Therefore, these non-overlapped areas lack metal melting and flow, though it is impossible to achieve “reducing the ridges and filling the valleys” effect. Therefore, whether the remelted passes overlap well is related to whether all the areas of the surface are fully remelted, affecting the improvement of flatness. Therefore, in production, the maximum remelting spacing should be set based on experimental results.

When the axis of the tungsten electrode points toward the ridge of the additive part’s surface, the stress and flow conditions of the molten pool have been analyzed in Section 3.1.2. However, when the remelting spacing is either larger or smaller, the tungsten electrode does not always align with the ridge line. When the tungsten electrode’s axis deviates from the center of the ridge, the center of the molten pool may appear on a slope or in a valley. In one of the scenarios, when the center of the molten pool is located in a valley, gravity draws the liquid metal from the slopes or ridges on either side toward the pool’s center, while buoyancy causes the center of the molten pool to flow upward. This distribution of the resultant force from gravity and buoyancy is opposite to that in the molten pool at the ridge, but both serve to flatten the surface of the molten pool. Compared to the surface tension effects in the molten pool at the ridge, the surface tension of the recessed molten pool surface creates additional pressure directed toward the curvature center (above the molten pool surface) [54], which promotes the flattening of the surface. The arc pressure at the center of the molten pool hinders the flattening of the molten pool surface, but the arc pressure from the TIG arc is small. Furthermore, due to the minimum arc voltage principle, the arc will automatically choose an appropriate current path to ensure the minimum electric field intensity [55]. Therefore, the anode spot of the arc may shift to the slopes on both sides of the valley, especially when the valley is relatively narrow, as shown in Figure 2 and Figure 8, causing the remelting current to split towards both slopes. The peak of the arc pressure may occur on the slopes. The resulting molten pool flows toward the valley center under the influence of gravity, filling the valley. In another scenario, when the center of the molten pool is on a slope, gravity, arc pressure, and the shear force from the plasma flow (drag effect) may all cause the molten metal to flow toward the valley. According to the discussion above, when the remelting spacing is 3 mm, although the tungsten electrode does not always directly face the ridge line, the remelting arc with sufficient current can still improve the flatness. Effective overlap between the remelted passes ensures improvement, although employing a smaller remelting spacing could potentially compromise process efficiency. At a remelting spacing of 5 mm, the improvement in flatness is also noticeable. However, due to poor overlap, the effect is relatively less markedly compared to 4 mm.

### 3.3. Changes in Surface Characteristics with Various Remelting Directions

**Figure 11:**
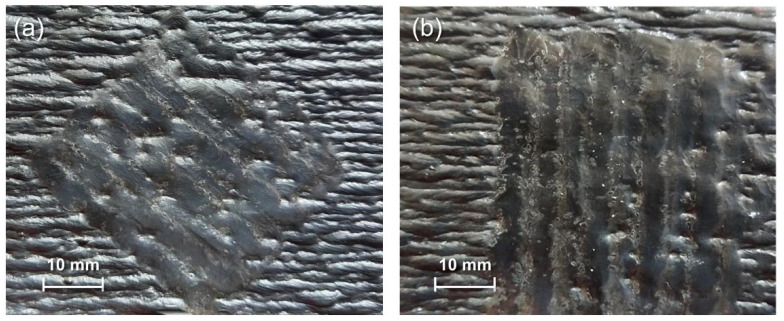
Surface morphology of remelted zones with different remelting directions: (**a**) 45 and (**b**) 90° to the original additive deposition direction.

**Figure 12:**
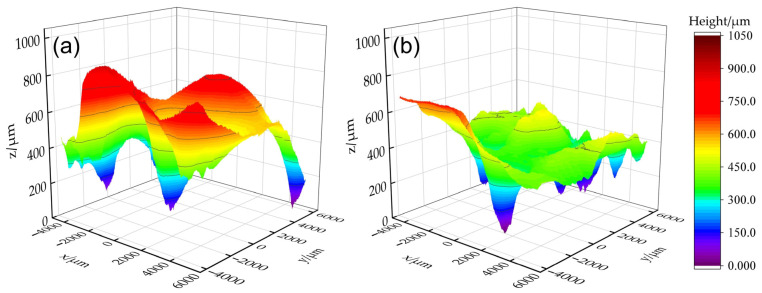
Three-dimensional reconstructed contours of the typically measured areas with remelting directions: (**a**) 45 and (**b**) 90° to the original additive deposition direction.

Another scenario that may occur in a production environment is when the remelting direction is not parallel to the deposition direction of the additive part. This section discusses whether, under such circumstances, TIGAR can still improve the surface flatness of additive parts. When the remelting direction is at 45° and 90° (with a remelting current of 80 A and other process parameters the same as in previous experiments, i.e., arc voltage of 14 V and remelting speed of 1 mm/s), the surface morphology is shown in Figure 11a,b, respectively. It can be observed that there are fluctuations in the width of the remelted passes, leading to poorly overlapped areas between adjacent passes—partially resulting in pits between adjacent remelted passes, which are more likely to appear in the “valleys” of the original additive part’s surface. The corresponding three-dimensional reconstructions in Figure 12 show that the surface height for the measured area of the 45° specimen (Figure 12a) is mostly in the green, yellow, and orange regions of 300~650 μm, with distinct red peak areas; whereas for the measured area of 90° specimen (Figure 12b), the surface height is mostly in the green region of 300~500 μm. The surface topography of the two cases above shows a certain degree of improvement relative to non-remelted regions (Figure 4a). But compared to the parallel remelting case (Figure 4d), the undulations are still significantly increased.

**Figure 13:**
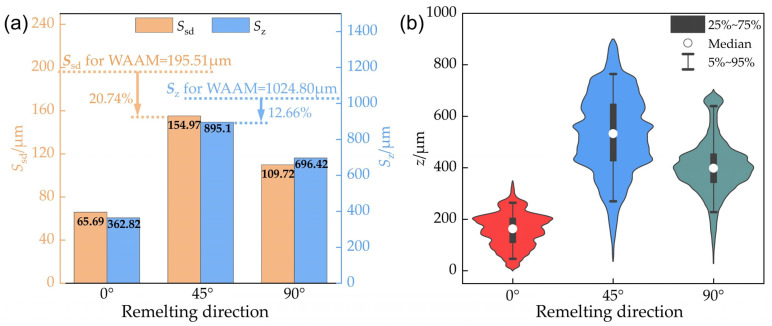
Characterization of surface morphology of measured areas with different remelting direction: (**a**) standard deviation of height (*S*_sd_) and maximum height difference (*S*_z_); (**b**) violin plots illustrating height distribution.

The standard deviation of height *S*_sd_ and the maximum height difference *S*_z_ of the measured areas in the figure above are shown in Figure 13a. When the remelting direction is at 45° and 90°, the *S*_sd_ values are 154.97 μm and 109.72 μm, respectively, which represent reductions of 20.74% and 43.89% compared to the non-remelted area. The *S*_z_ values are 895.1 μm and 696.42 μm, respectively, which represent reductions of 12.66% and 32.04% compared to the non-remelted area. When the remelting direction is at 45°, both the *S*_sd_ and *S*_z_ show small improvement margins compared to the non-remelted area, with *S*_z_ increasing by 532.28 μm and *S*_sd_ increasing by 89.28 μm compared to the parallel remelting specimen. As illustrated in Figure 13b, the height distribution of the measured area indicates that the IQR in height for the 45° remelting specimen is 220.18 μm, significantly larger than that for parallel remelting (114.51 μm), and the height median is also markedly higher. Although the 90° case has a similar IQR compared to that of the parallel remelting case, there are more points with height values significantly deviating from the median, forming distinct red peaks in Figure 12b. Consequently, its height standard deviation *S*_sd_ is 67% larger than in the parallel case. The analysis above indicates that compared to parallel remelting, remelting perpendicular or at a 45° angle to the original additive weld beads significantly reduces the effectiveness of flatness improvement.

Based on the analysis in Section 3.2, it is evident that regardless of whether the tungsten electrode centerline is aligned with the ridges, valleys, or even slopes, the arc has an effect of flattening the surface of the additive part. So why is the improvement significantly reduced in the experiments of this section? When the remelting path forms a 45° and 90° angle with the deposition direction of the additive part, the centerline of the tungsten electrode passes through alternating ridges and valleys, with the arc length constantly changing, leading to slightly uneven heat input along the remelting path. This results in insufficient melting in some areas, and intermittent or fluctuating existence of the molten pool. The difficulties in the metal-fluid flow over certain locations where the heat input is lower significantly reduced the “valleys filling” effect.

**Figure 14:**
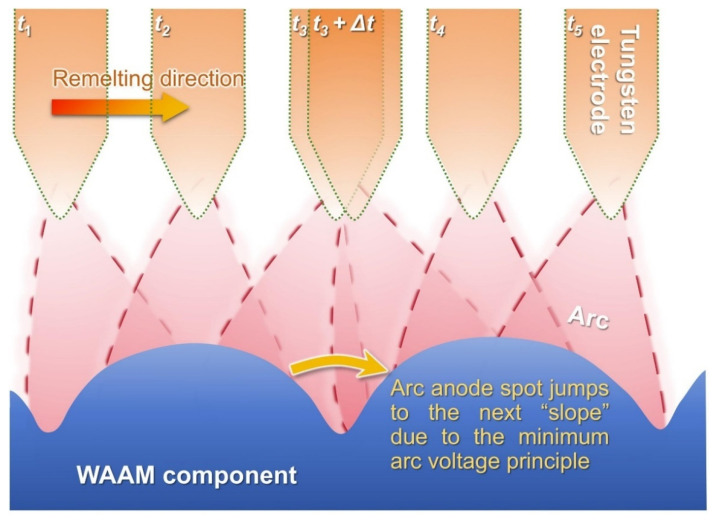
Schematic diagram of the arc jumping phenomenon (*t*_1_~*t*_5_ are five equally spaced moments, while Δ*t* is a small-time difference).

Moreover, according to the principle of minimum voltage, the arc will automatically seek an appropriate current path to ensure a minimum electric field intensity (in a uniform medium, usually the shortest path in geometry) to keep the voltage as low as possible. As the welding torch moves forward, the point on the additive part’s surface closest to the tungsten tip also moves forward. When the surface of the additive part has notable undulations, this point might skip over the valley, jumping directly from one slope to the opposite slope. Therefore, the principle of minimum voltage may cause the anode spot to bypass the lowest point of the valley, as shown in Figure 14. These phenomena ultimately lead to uneven surface improvement of the additive part, with some valleys not being adequately filled with liquid metal. The slightly better surface flatness improvement of the 90° specimen compared to the 45° specimen may be related to the shorter ridge spacing on its remelting path and shorter arc jumping distances.

Obviously, in actual production processes, there would not be such significant directional deviations. It can be inferred that slight deviations from the additive deposition direction during remelting will not have such a pronounced reduction in flatness improvement. When parallel or nearly parallel to the original additive part’s deposition direction, the deviation of the tungsten electrode centerline from the ridge changes only slightly over time, with the arc length changing slowly, and the shortest conductive path may not jump due to the extremely slow variation in undulations. Based on the discussions in Section 3.1 and Section 3.2, it is speculated that at small angles, a significant reduction in remelting effects can be avoided.

In summary, the remelting direction has a noticeable impact on the effectiveness of the TIGAR process at a large directional deviation, and it is better for remelting to be conducted as parallel as possible to the original additive deposition path.

### 3.4. Comparison of the Surface Improving Methods from Existing Research

In this section, the authors compare different methods for improving surface flatness, briefly listing their advantages and limitations, as well as their processing efficiency, post-processing surface quality, and costs, as shown in Table 4. Those methods were mentioned in the introduction in this paper. Since all the methods, except for machining, are still in the laboratory research stage, and even the WAAM technology has not been widely applied on a large scale. Therefore, the processing efficiencies in the table are based on experimental parameters and do not represent the actual industrial production efficiency, nor do they take into account the time required for process alternations. The simplified processing efficiency of machining can be calculated via Equation (10),

(10) $$E_{M}=\frac{a_{e}f}{H/a_{p}}$$

where *E*_M_ is the efficiency, also the machined area per second, *a*_e_ is radial feed, *f* is feed rate, *H* is the thickness of the whole cutting layer, *a*_p_ is the cutting depth (axial feed). The result of $H/a_{p}$ should be rounded up to the nearest whole number.

And the processing efficiency of remelting *E*_R_ can be calculated via the equation below,

(11) $$E_{R}=D_{R}\nu_{R}$$

where *D*_R_ is the remelting spacing, *v*_R_ is the remelting speed.

As can be seen from Table 4, in existing research, the TIGAR method can process complex part surfaces and large-scale parts, achieving surface flatness close to that of laser remelting, but at a cost far lower than laser remelting and machining methods. For construction components, art installations, and equipment parts with low esthetic and assembly requirements, the precision of the TIGAR process can meet their accuracy demands. Since the TIG welding torch does not require wire feeding and is highly flexible and lightweight, it is particularly suitable for the processing of super-large structures that exceed the working range of machine tools and positioners. However, there is still room for improvement in its processing efficiency; from an intuitive experience, its processing speed is lower than that of machining and rolling, but machining may require multiple layers of cutting. Another limitation is that the surface flatness after processing is still relatively insufficient. The TIGAR method makes it almost impossible to achieve the surface flatness of machining, and it cannot completely replace machining, especially for the manufacturing of mechanical equipment and devices.

### 3.5. Microstructure and Hardness of Remelted Surface

**Figure 15:**
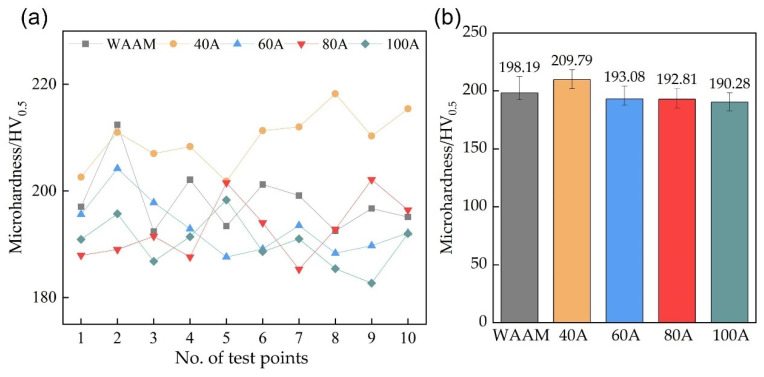
Vickers hardness values of (**a**) the WAAM parts surface and the remelting zones under various remelting currents, and (**b**) their means.

The aforementioned experiments have demonstrated that the TIGAR technology can improve the surface morphology of WAAM components. This section examines the impact of this process on the microstructure and surface hardness of additive parts. The microstructure and hardness of the additive part’s surface affect its service performance. Moreover, for parts that still require machining post-processing, surface hardness determines the difficulty and cost of machining. Ten randomly selected points were taken on the WAAM additive parts surface and in each remelting zone under various remelting currents, respectively, and the measured Vickers hardness is shown in Figure 15a, with the average values in Figure 15b. The average hardness of the remelted surface with a remelting current of 40 A, slightly increased, differing from the average hardness of additive part surface by only 5.9%. Under other remelting currents, the hardness slightly decreased, with the lowest surface hardness under a remelting current of 100 A, but it only decreased by 4% compared to the hardness of the WAAM specimen. The impact of TIGAR on the surface hardness is minimal, and its effect on service performance and machining post-processing difficulty can be neglected.

**Figure 16:**
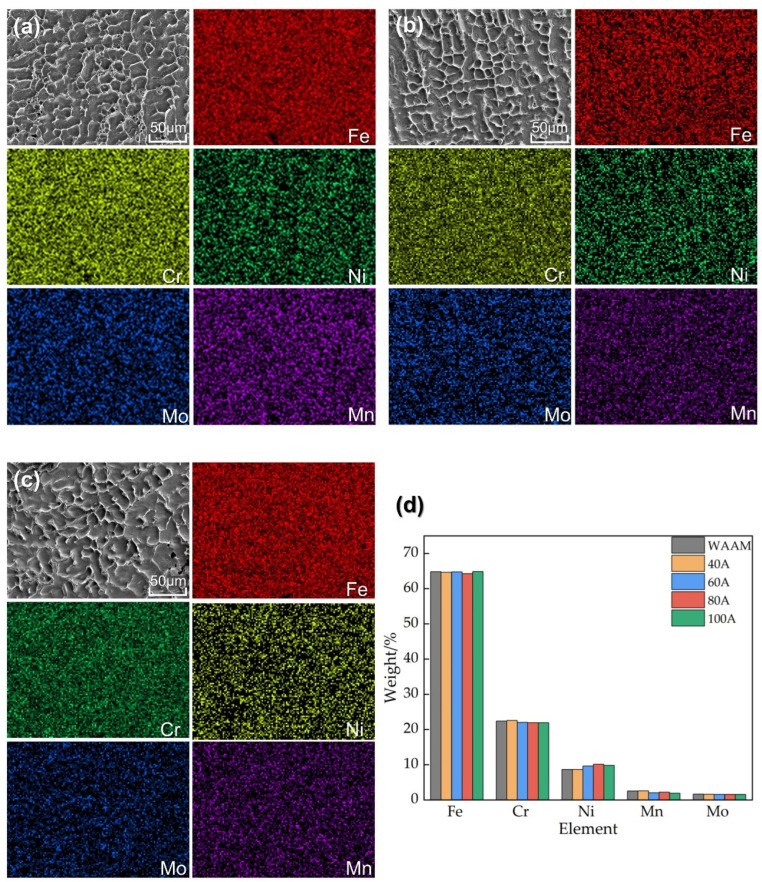
SEM morphology and the mapping results of the main elements of the near-surface areas (0.6 mm below the surface of each specimen) of (**a**) WAAM parts, (**b**) the remelting zone under 40 A, (**c**) the remelting zone under 100 A, and (**d**) the elemental contents diagram.

Microstructure observation can explain why there are minimal changes in performance. The SEM images and EDS mapping results of the near-surface areas (0.6 mm below the surface of each specimen) of the WAAM part and the remelted zones under the minimum remelting current (40 A) and the maximum remelting current (100 A) are shown in Figure 16a–c, respectively. It can be observed that the grain size and morphology are similar before and after remelting. During the TIGAR process, no new material was added, and the distribution and overall proportion of the five main elements (Fe, Cr, Ni, Mn, and Mo) in 316 L stainless steel (Figure 16d) only show slight differences, which are close to the composition of the original welding wire. No significant segregation is observed in Figure 16a–c. This indicates that the impact of remelting on the surface microstructure of additive parts is extremely limited.

## 4. Conclusions

Experiments were conducted to improve the morphology and flatness of the undulating surface of wall-shaped wire-arc additive manufactured 316 L parts using the TIGAR method. Abundant results were obtained, and detailed discussions were conducted. The following main conclusions were drawn:

1. The improvement effect on surface flatness is enhanced as the remelting current increases. When the current is set to 80 A, TIGAR can effectively reduce the maximum height difference in the surface by 65%, the standard deviation of surface height by 66%, and the cutting waste by 72%.
2. The analysis of the forces and flow within the molten pool explains this counterintuitive phenomenon—regardless of whether the axis of the tungsten electrode is aligned with the ridges or valleys of the original additive surface, the arc can flatten the surface. This characteristic ensures that when there is some deviation in the remelting spacing, such as in a rough production environment, the TIGAR process demonstrates robustness and adaptability.
3. The proper overlap of remelted passes is a critical condition for effective improvement in flatness. When the remelting spacing is too large, or when the deviation in the arc’s trajectory from the WAAM deposition direction leads to variations in the molten pool size, the resulting poor overlap can negatively affect the surface improvement.
4. The TIGAR process has a minimal impact on the surface microstructure and hardness of the additive part, which does not affect service performance and the machining post-processing, if necessary.
5. The TIGAR process shows great potential for enhancing surface quality of WAAM parts due to its low cost. However, there is still room for improvement in its processing efficiency. Another key limitation is that the surface flatness after processing is still far from the precision achieved by machining.

## 5. Outlook for Future Research

The TIGAR method still has many limitations. In subsequent research, the following work should be carried out to continue improving the process and promote its application:

1. Enhancing process efficiency by employing multiple tungsten electrodes or special-shaped electrodes to melt a wider width or area of the deposited surface of WAAM parts within the same unit of time would be valuable.
2. Investigating the suitable remelting process parameters for additive parts’ surfaces of different types or in different orientations would make the process more feasible.
3. Discussing the potential of combining TIGAR with other post-processing techniques or adapting the method to new materials could broaden its applicability.

## Figures and Tables

**Table 1:** Chemical composition of 316 L stainless steel (wt%).

Element	Cr	Ni	Mo	Mn	Si	P	S
Content (wt%)	18.0~20.0	10.0~14.0	2.0~3.0	1.0~2.5	≤1.0	≤0.045	≤0.030

**Table 2:** Principle parameters of the WAAM process.

Welding Current (A)	Arc Voltage (V)	Travel Speed (mm/s)	Inter-Layer Cooling Time (s)	Number of WAAM Layers
120	18.5	5	60	30

**Table 3:** Process parameters of the TIGAR experiments.

Test No.	Remelting Current (A)	Arc Voltage (V)	Travel Speed (mm/s)	Remelting Spacing (mm)	Remelting Directions
A1	40	14	1	4	0°
A2	60	14	1	4	0°
A3	80	14	1	4	0°
A4	100	14	1	4	0°
B1	80	14	1	3	0°
B2	80	14	1	5	0°
C1	80	14	1	4	45°
C2	80	14	1	4	90°

**Table 4:** Comparison of different methods for improving surface flatness.

Methods	Machining	Laser Remelting	TIGAR	Controlling the WAAM Process	Rolling
Advantages	High surface quality	Convenient for regulating the heat source	Low cost Robust as discussed in Section 3.2 Similar surface quality as expensive laser remelting method Flexibility and adaptability	No additional processes or time other than WAAM itself are required.	1. It can improve microstructure and properties; 2. It is more suitable for eliminating deformation of parts.
Limitations	1. It cannot be easily applied to large-scale components, as discussed in the 5th paragraph of Section 1. 2. It produces cutting waste.	1. Difficult to make the surface look “flat” to the naked eye 2. Expensive	Difficult to make the surface look “flat” to the naked eye	It is hard to eliminate the undulating profile with alternating ridges and valleys on the surface of the WAAM parts because the inherent characteristics of the WAAM process.	1. It Is hard to be applied to complex shapes that involve close features and intersections; 2. It cannot be easily applied to large-scale components; 3. It is difficult to make the surface look “flat” to the naked eye, (for the cooled sides of wall parts).
Efficiency	Calculated via Equation (10): NG × 16/(*H*/0.3) mm^2^/s [51]; NG × 0.2/(*H*/0.5) mm^2^/s with thermal milling [50]; 2.4 × 0.83/(*H*/0.4) mm^2^/s [52]. (NG means “Not Given”.)	Similarly to or faster than TIGAR	Calculated via Equation (11): 1 × 4 mm^2^/s	No additional time other than WAAM itself, but some methods, such as utilizing lower current, will decrease the WAAM efficiency.	Faster than remelting, but there is a lack of specific data on the processing efficiency.
Post-processing quality (the maximum height difference in surface)	In the range of several micrometers	Similarly to TIGAR [31,32]	Less than 300 micrometers	In the range of several millimeters	In the range of several hundred micrometers (1~2 hundred micrometers for hot rolling [30])
Cost	Operating power: tens of kilowatts	Cost for equipment: tens of thousands of dollars Operating power: several kilowatts	Cost for equipment: tens of thousands of dollars Operating power: 1~2 kilowatts	0~thousands of dollars	Cost for equipment: thousands of dollars Operating power: several kilowatts

## Data Availability

The original contributions presented in this study are included in the article. Further inquiries can be directed to the corresponding author.
